# Nucleoid localization of Hsp40 Mdj1 is important for its function in maintenance of mitochondrial DNA^☆^

**DOI:** 10.1016/j.bbamcr.2013.05.012

**Published:** 2013-10

**Authors:** Grzegorz L. Ciesielski, Magdalena Plotka, Mateusz Manicki, Brenda A. Schilke, Rafal Dutkiewicz, Chandan Sahi, Jaroslaw Marszalek, Elizabeth A. Craig

**Affiliations:** a Department of Molecular and Cellular Biology, Faculty of Biotechnology, University of Gdansk, 80822 Gdansk, Poland; b Department of Biochemistry, University of Wisconsin–Madison, Madison, WI 53706, USA

**Keywords:** J-protein, Molecular chaperone, DNA transactions, Yeast

## Abstract

Faithful replication and propagation of mitochondrial DNA (mtDNA) is critical for cellular respiration. Molecular chaperones, ubiquitous proteins involved in protein folding and remodeling of protein complexes, have been implicated in mtDNA transactions. In particular, cells lacking Mdj1, an Hsp40 co-chaperone of Hsp70 in the mitochondrial matrix, do not maintain functional mtDNA. Here we report that the great majority of Mdj1 is associated with nucleoids, DNA-protein complexes that are the functional unit of mtDNA transactions. Underscoring the importance of Hsp70 chaperone activity in the maintenance of mtDNA, an Mdj1 variant having an alteration in the Hsp70-interacting J-domain does not maintain mtDNA. However, a J-domain containing fragment expressed at the level that Mdj1 is normally present is not competent to maintain mtDNA, suggesting a function of Mdj1 beyond that carried out by its J-domain. Nevertheless, loss of mtDNA function upon Mdj1 depletion is retarded when the J-domain, is overexpressed. Analysis of Mdj1 variants revealed a correlation between nucleoid association and DNA maintenance activity, suggesting that localization is functionally important. We found that Mdj1 has DNA binding activity and that variants retaining DNA-binding activity also retained nucleoid association. Together, our results are consistent with a model in which Mdj1, tethered to the nucleoid *via* DNA binding, thus driving a high local concentration of the Hsp70 machinery, is important for faithful DNA maintenance and propagation.

## Highlights

• J-protein co-chaperone Mdj1 is critical for maintenance of functional mtDNA.
• Majority of Mdj1 localizes to the nucleoid, likely *via* interaction with mtDNA.
• Nucleoid localization of Mdj1 is necessary for mtDNA maintenance.
• Function of Mdj1 in mtDNA maintenance requires cooperation with Hsp70.

## 1 Introduction

Mitochondria are essential organelles required for key cellular functions, including ATP production by oxidative phosphorylation. As several of the proteins required for oxidative phosphorylation are encoded by the mitochondrial genome, efficient replication and transmission of mitochondrial DNA (mtDNA) is critical [1]. In humans a number of diseases have been linked not only to mutations in mtDNA itself, but also to mutations in nuclear genes encoding proteins involved in maintenance and propagation of the mitochondrial genome [2]. The yeast *Saccharomyces cerevisiae* has served as a useful model for the understanding of mitochondrial functions, particularly the maintenance and propagation of the mitochondrial genome [3,4]. The fact that yeast cells lacking functional mtDNA are viable as long as a fermentable carbon source such as glucose is provided has proven particularly advantageous.

In all eukaryotes, mtDNA is assembled into nucleoprotein complexes called mitochondrial nucleoids, the functional unit of mtDNA propagation, segregation and expression [1,5]. In *S. cerevisiae*, each nucleoid contains several genome equivalents of mtDNA, with each cell containing approximately 40 nucleoids [6]. A diverse group of proteins has been found to be associated with nucleoids in fungi, vertebrates and plants [6,7]. Some of these nucleoid proteins, such as mtDNA polymerase or mitochondrial helicases, have enzymatic activities known to be critical for mtDNA maintenance and propagation. Others, such as Abf2 of *S. cerevisiae*, an HMG DNA-binding family member related to nuclear chromosomal proteins, are devoid of any apparent enzymatic activity, but rather are involved in the packaging of mtDNA [8]. Germane to this report, molecular chaperones, in particular the Hsp70 system, have also been linked to mitochondrial nucleoids and the stability of mtDNA [5,6,9,10]. The mammalian 40 kDa J-protein co-chaperone of Hsp70, DnajA3 (also known as Tid1), has been found associated with mitochondrial nucleoids [9]. Mice deficient in DnajA3 develop dilated cardiomyopathy and have reduced amounts of mtDNA [11]. In *S. cerevisiae*, deletion of *MDJ1*, the DnajA3 ortholog, leads to loss of functional mtDNA [12–14].

Hsp70 chaperone systems are ubiquitous. *Via* their interactions with client proteins they play roles in the prevention of protein aggregation, folding of newly synthesized and partially denatured proteins, and remodeling of protein:protein complexes [15,16]. Like other J-proteins, Mdj1 plays the critical role of stimulating the ATPase activity of its partner Hsp70 (Ssc1), thus stabilizing client protein interaction with Hsp70 [17]. The defining feature of all J-proteins, including Mdj1, is a ~ 70 amino acid J-domain, which is directly responsible for this stimulation. Mdj1 has a complex architecture, very similar to that of other so-called Hsp40s or Class I J-proteins, such as DnaJ of *Escherichia coli* and Ydj1/DnajA1 of the yeast/mammalian cytosol [18,19]. Immediately adjacent to the N-terminal J-domain of Class I J-proteins is a glycine/phenylalanine (GF)-rich linker region, followed by the client protein binding region, which is composed of two barrel topology domains, CTD1 and CTD2. CTD1 has a hydrophobic pocket shown to bind client peptides in this class of J-proteins, as well as the zinc finger-like domain extruding from it, which also may be involved in client binding [20–22]. The extreme C-terminus is a dimerization domain.

This structural complexity allows Mdj1 and other members of this class of J-proteins to function in diverse roles, by binding to client proteins and “delivering” them to Hsp70 [15,16]. The Mdj1/Hsp70 machinery has been shown to prevent aggregation and participate in reactivation of mitochondrial proteins, including DNA polymerase [23–25]. But this protection of mtDNA polymerase appears to be important only under stress conditions such as heat shock or during growth at borderline temperatures [13,24]. On the other hand, the requirement of Mdj1 function for maintenance of functional mtDNA appears absolute [12,13]. While mutants lacking other mitochondrial nucleoid proteins such as Abf2 maintain functional mtDNA, if “forced” to grow on non-fermentable carbon sources [26], respiratory competent *mdj1*-∆ cells have not been obtained even under such circumstances.

To better understand Mdj1's critical role(s) in the maintenance of the mitochondrial genome, we developed a system to follow the kinetics of loss of mtDNA function upon depletion of Mdj1. Here we report that, not only is the co-chaperone function of Mdj1, that is the ability to stimulate its partner Hsp70's ATPase activity, essential for mtDNA maintenance, a second activity is required as well. The ability of Mdj1 to bind DNA correlated with its ability to efficiently function in mtDNA maintenance, is consistent with its localization to the mitochondrial nucleoid being due to its DNA binding ability.

## 2 Material and methods

### 2.1 Plasmids

*TETrMDJ1* was created by insertion of the *MDJ1* open reading frame into the vector pCM189 [27], placing it under control of a tetracycline-regulated promoter (*TETr*). Unless otherwise indicated, other plasmids used in this study are based on the pRS plasmid series [28]. *MDJ1* was obtained by PCR amplification of genomic DNA from chromosome VI position 94695 to 116230 and cloned into pRS316 (*URA3 CEN*) or pRS313 (*HIS3 CEN*) vectors. Mutants of *MDJ1* were constructed by site-directed mutagenesis: Mdj1_H89Q_, His^89^ replaced by Gln; Mdj1_LFI/AAA_, Leu^222^ Phe^224^ Ile^301^ replaced by Ala; Mdj1_Δ190–511_, C-terminal deletion of residues 190 to 511; Mdj1_∆J_ deletion of residues 55 to 123; Mdj1_ΔC_, internal deletion of residues 190 to 429; Mdj1_ΔD_, C-terminal deletion of residues 430 to 511; Mdj1_ΔZ_, internal deletion of residues 230 to 288; Mdj1_H89Q∆D_, His^89^ replaced by Gln and C-terminal deletion of residues 430 to 511; Mdj1_ΔZ∆D_, internal deletion of residues 190 to 429 and C-terminal deletion of residues 430 to 511. Plasmids for overexpression of wild-type Mdj1 or *mdj1*_Δ190–511_ were obtained by insertion of appropriate open reading frames into pRS416*TEF* and pRS413*TEF* vectors [29]. For fluorescence microscopy studies, *MDJ1-GFP* and mutant *mdj1-GFP* fusion genes were inserted into pRS416*TEF* vector. Immunoblot analysis demonstrated that the fusion proteins were intact in yeast cells (data not shown).

For Mdj1 protein purification, plasmid pBAD22A *MDJ1*Δ55 *HIS_6_* was constructed by addition of PCR generated fragments encoding 6xHis-tag at the 3′ of *MDJ1*Δ55 (which lacks the codons for the mitochondrial targeting sequence, residues 1 to 55) into the pBAD22A vector [17]. For purification of the Mdj1 variants listed above, pBAD22A plasmids harboring these mutants were created by site-directed mutagenesis, using pBAD22A *MDJ1*Δ55 *HIS_6_* as a template. For visualization of Abf2, yeast cells were transformed with a plasmid encoding the *ABF2-GFP* fusion, pRS416 *GAL1 ABF2-GFP*. For visualization of mitochondria, yeast cells were transformed with a plasmid encoding mitochondria-targeted GFP (mtGFP), pRS416 *GAL1 mt-GFP* (both kindly provided by Dr. Benedikt Westermann, University of Bayreuth, Germany).

### 2.2 Yeast strains, media and chemicals

All strains used had the W303 genetic background (PJ51-3a): MAT a *trp1-1 ura3-1 leu2-3*,*112 his3-11*,*15 ade2-1 can1-100 GAL2 met2-1 lys2-2* (James P and Craig EA, unpublished data). A strain having Mdj1 expression under the control of a dual tetracycline regulated system, called *mdj1*-Δ [*TETr-MDJ1*], was derived from diploid PJ53 *MDJ1*/*mdj1::TRP1* by integration of a linearized pCM244 plasmid [30] encoding a repressor gene (*TETr′-SSN6*) under control of the CMV promoter at the chromosomal *LEU2* locus (CMVp(*TETr′-SSN6*)::*LEU2*) and transformation with the *TETrMDJ1* plasmid, followed by tetrad dissection. A TRP^+^URA^+^LEU^+^ haploid strain was obtained by tetrad dissection. For Mdj1 localization studies utilizing fluorescence microscopy and sucrose gradient centrifugation analysis, a diploid derivative PJ53 *MDJ1*/*mdj1::TRP1* was used. Yeast cells were grown on YPD (1% yeast extract, 2% peptone and 2% glucose), YPG (1% yeast extract, 2% peptone and 3% glycerol) or synthetic media as described in [31]. All chemicals, unless stated otherwise, were purchased from Sigma.

### 2.3 Fluorescence microscopy

Yeast cells (PJ53 *MDJ1*/*mdj1::TRP1*) expressing plasmid borne copies of *mt-GFP* and GFP-fusion proteins were grown to log phase on glycerol-containing synthetic media. Expression of Abf2-GFP and mt-GFP under control of a *GAL*-promoter was induced by galactose (1% w/v) addition to the medium. *mdj1-GFP* variants were constitutively expressed under the *TEF* promoter. mtDNA was stained by incubation with 0.5 μg/ml 4′,6-diamidino-2-phenylindole (DAPI) for 10 min. The mitochondrial network was stained by incubation for 30 min with MitoTracker Red CMXRos (Invitrogen Molecular Probes) at 40 nM final concentration. Harvested cells were immobilized on microscope slides with 1% agarose and viewed under an Olympus BX51 upright microscope with an oil-immersion 100 × PlanApo 1,4 NA objective. GFP fluorescence was viewed with the GFP filter set (451–490 nm excitation filter; 495–540 band pass emission filter), and blue fluorescence of DAPI stained nucleoids was recorded with the DAPI filter set (360–370 nm excitation filter; > 420 band pass emission filter). Images were taken by an F-View II (Soft Imaging System GmbH) CCD camera operated *via* analySIS® image analysis software.

### 2.4 Mitochondrial nucleoid complex isolation by sucrose gradient centrifugation

Mitochondria were isolated as described previously [32]. Linear 35–70% sucrose gradients were prepared in buffer SG (20 mM Tris–HCl pH 7.6, 1 mM ethylenediaminetetraacetic acid (EDTA), 7 mM β-mercaptoethanol, 1 mM spermidine and 1 mM phenylmethylsulfonyl fluoride (PMSF)) in 3 ml test tubes, frozen on dry-ice, stored at − 70 °C, and before use left for one hour in the cold room to thaw. Isolated mitochondria (1 mg protein) were suspended in 0.7 ml of buffer SG and lysed in the presence of 0.5% (v/v) NP-40, 1 mM PMSF and 3 mM spermidine. The mixture was kept on ice for 15 min. The lysis conditions used were such that mitochondrial membranes were fully solubilized, for details see [35]. Mitochondrial lysates were loaded onto the top of the gradient and centrifuged in a Beckman SW60Ti rotor for 1 h, 46,000 ×*g* at 4 °C. Following centrifugation, fractions (233 μl) were collected from the top of the gradient for further analysis. Each fraction was analyzed for mtDNA content by PCR amplification of mtDNA fragment *ori5* (281 bp; 82326…82606 mtDNA). The PCR conditions used were such that the amount of PCR product was proportional to the amount of the mtDNA present in the sample, as indicated by the amount of PCR product detected in each sample correlating with its absorbance measured at 260 nm and that sample dilution resulted in appropriately reduced amounts of PCR product.

### 2.5 Assay for maintenance of respiratory competence upon MDJ1 repression

All cell cultures used for *MDJ1* repression assays were grown at 30 °C in liquid synthetic glucose containing media necessary to maintain non-essential plasmids. Cells were maintained in exponential growth phase by continual subculturing into fresh liquid media. *MDJ1* repression time-course assays were carried out in the absence or presence of 5 μg/ml of doxycycline. For analysis of respiratory competence, aliquots were collected and serially diluted such that approximately 100 cells were plated on YPD (for color differentiation) or YPG (to directly assess ability to respire). To examine Mdj1 cellular protein levels, cells equal to one OD_600_ were harvested and stored at − 70 °C prior to immunoblotting analysis. For densitometry analysis ImageJ software was used [33].

### 2.6 Isolation and analysis of mtDNA

For the purification of mtDNA, total cellular DNA was isolated. mtDNA was separated from nuclear DNA essentially as described previously [34]. Briefly, 0.5-liter cultures were grown to OD_600_ of 2.0 to 3.0 and the cells were harvested by centrifugation. After treatment with zymolyase, the resulting spheroplasts were washed with 100 ml of 1.2 M sorbitol, resuspended in 100 ml of 20 mM Tris–HCl pH 8, 50 mM EDTA, 1% (w/v) sodium dodecyl sulfate (SDS), and incubated for 30 min at 65 °C. Then 50 ml of 5 M potassium acetate was added and incubated on ice for 45 min. After centrifugation at 9000 ×*g* for 10 min, 150 ml of isopropanol was added to the supernatant. Precipitated DNA was resuspended in 10 ml of 10 mM Tris–HCl (pH 8.0), 1 mM EDTA. Then, 1.16 g of CsCl for 1 ml of DNA solution and 24 μl of (10 mg/ml) bisbenzimide (Hoechst 33258; Sigma) were added. The mixture was centrifuged in an NVT 90 rotor (Beckman Instruments) for 16 h at 65,600 rpm. DNA was visualized with long-wavelength UV light. Standard methods were used for the analysis of isolated mtDNA with the restriction enzyme EcoRV.

### 2.7 Protein purification

*E. coli* pOD259 [17] was transformed with pBAD22A harboring either *MDJ1*Δ55 *HIS_6_* or one of the *mdj1*Δ55 *HIS_6_* variants. A 6 liter culture was grown at 30 °C in LB broth supplemented with ampicillin (100 μg/ml). At an OD_600_ of 0.7 arabinose was added to the final concentration of 0.2% (w/v) and the culture was grown for another 2 h. Cells were harvested by centrifugation, resuspended in 20 ml of buffer S (25 mM HEPES pH 7.5, 200 mM KCl, 10% (v/v) glycerol, and 1 mM PMSF) and lysed in the presence of 1 mg/ml lysozyme for 45 min on ice. During the last 15 min, Brij 58 was added to a final concentration of 0.6% (v/v). Subsequently, cells were sonicated for 3 min, 30 s on/off bursts, 70% power (Misonix Sonicator 3000). The lysate was centrifuged in a Beckman JA 30.50 rotor at 25,000 rpm (75600 ×*g*) for 30 min and the supernatant with the addition of 50 mM imidazole was loaded onto a 1 ml Ni-NTA agarose column, previously equilibrated with buffer A (25 mM HEPES pH 7.5, 200 mM KCl, 10% (v/v) glycerol, 1 mM PMSF, 0.05% (v/v) Brij 58, and 50 mM imidazole). The column was subsequently washed with 20 volumes of buffer A, 20 volumes of buffer A with 0.6 M KCl and 20 volumes of buffer A with 1 mM ATP. Proteins were eluted with steps of 250 mM imidazole in buffer E (25 mM HEPES, pH 7.5, 50 mM KCl, 10% (v/v) glycerol, 0.05% (v/v) Brij 58, and 5 mM β-mercaptoethanol). 1 ml samples were collected and stored at − 70 °C. To avoid precipitation during dialysis, fractions containing highly concentrated protein were directly mixed with 300 μl of Source Q resin equilibrated with buffer Q (25 mM HEPES pH 7.5, 50 mM KCl, 10% (v/v) glycerol, 0.05% (v/v) Brij 58, and 1 mM DTT) in a 15 ml corning tube. After 1 h of incubation with constant mixing the suspension was centrifuged. Next, the resin was washed with 10 volumes of buffer Q and transferred to an eppendorf tube after resuspending in 2 volumes of elution buffer (0.2 M KCl in buffer Q). Separated supernatant was stored at − 70 °C.

### 2.8 Expression levels of Mdj1 variants

To quantify expression levels of Mdj1 variants total protein extracts were prepared from 1 ml (OD_600_ = 1) of cells after growth in the presence of doxycycline for 48 h. Proteins were separated by SDS-PAGE in 12.5% or 15% gel and transferred to PVDF membrane (Immobilon-P^SQ^) in BSN transfer buffer (48 mM Tris, 39 mM glycine, 20% methanol, 1.3 mM SDS) for 1 h at 100 V. The membrane was washed in TBS buffer (50 mM Tris–HCl pH = 7.4, 200 mM NaCl), blocked in 3% milk for 30 min, washed again in TBS buffer with 0.1% Tween 20 and incubated O/N with antibodies specific to Mdj1 or Porin. Because the polyclonal antibody used reacts differently with wt and truncated forms of Mdj1, 0.2 pmol of purified wt Mdj1 or variants was run on the same gel, as a quantity reference for expression. Protein concentration was determined with SYPRO-Ruby protein stain (Molecular Probes, Eugene, OR) using bovine serum albumin as a standard.

### 2.9 DNA binding *in vitro*

*ori5* linear DNA fragment (71 bp; 82536…82606 mtDNA) used for DNA binding *in vitro* was prepared as described previously [35]. Purified Mdj1 protein and γ-^32^P-labeled *ori5* DNA were mixed and processed as described previously [35].

## 3 Results

### 3.1 Mdj1 localizes to the mitochondrial nucleoid

**Fig. 1:**
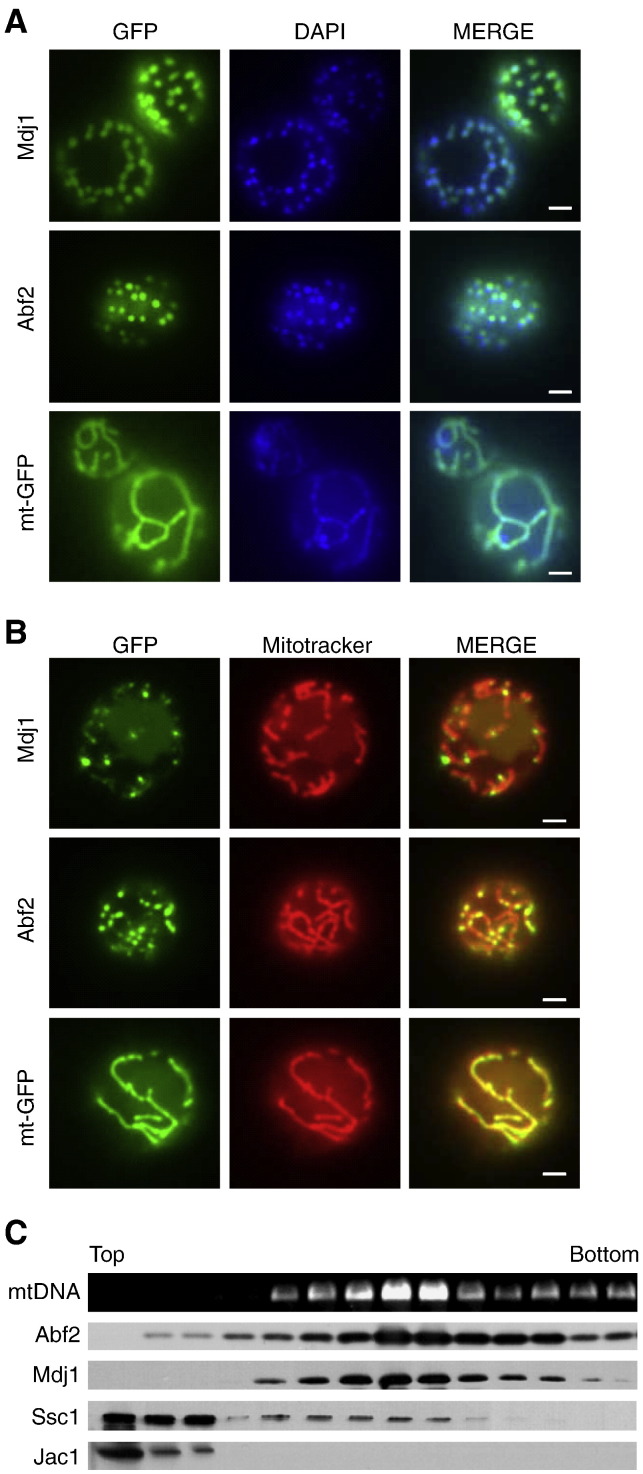
Mdj1 localizes to mitochondrial nucleoid. (A,B) *In vivo* localization of Mdj1. Wild-type (wt) cells expressing Mdj1-GFP, Abf2-GFP or mt-GFP were analyzed by fluorescence microscopy. Images in the two right-most panels were overlaid (MERGE). Size bars (2 μm) are shown. (A) Cellular DNA was stained using DAPI. (B) Mitochondrial network was stained using MitoTracker. (C) Distribution of Mdj1 after centrifugation of mitochondrial lysates. Lysates prepared from isolated mitochondria were subjected to ultracentrifugation through a sucrose gradient. A portion of each fraction was analyzed for mtDNA content by PCR amplification of mtDNA *ori5* fragment (mtDNA) and for protein content by immunoblot analysis using antibodies specific for Mdj1, Abf2, Ssc1 and Jac1.

Considering that Mdj1 is necessary for maintenance of functional mtDNA and that the human Mdj1 ortholog (DnajA3/Tid1) has been detected associated with the mitochondrial nucleoid complex [9], we decided to determine the localization of Mdj1 within the yeast mitochondrial matrix. We used two approaches, one microscopic, visualizing Mdj1 in living cells and, the second, biochemical, fractionating mitochondrial extracts by sucrose gradient centrifugation. To visualize Mdj1 we constructed a chimeric gene to allow expression of a fusion between Mdj1 and green fluorescent protein (GFP). Mdj1-GFP supported maintenance of mtDNA and rescued the growth defect of *mdj1*-Δ cells, demonstrating the functionality of the fusion protein (Suppl. Fig. S1). As positive and negative controls, we tested a GFP fusion of Abf2, a major core packaging protein of the mitochondrial nucleoid, and GFP itself, targeted to the mitochondrial matrix by the presence of a fused N-terminal cleavable pre-sequence (mt-GFP), respectively (Fig. 1A). As expected [1], Abf2-GFP was concentrated in distinct, dot-like structures, which co-localized with DAPI stained mtDNA. On the other hand, mt-GFP showed a diffuse pattern throughout mitochondrial tubules [36]. Mdj1-GFP fluorescence showed a very similar pattern to that of Abf2-GFP, concentrated in distinct, dot-like structures, which co-localized with DAPI stained mtDNA (Fig. 1A, B).

For biochemical analysis, extracts of mitochondria were subjected to centrifugation through sucrose gradients [35], thus separating mtDNA and associated proteins from soluble proteins. Under the conditions used, mtDNA and associated proteins such as Abf2, migrate to the middle of the gradient, while soluble matrix proteins such as the J-protein, Jac1, involved in the biogenesis of proteins containing iron-sulfur cluster prosthetic groups [37], remained at the top (Fig. 1C). The vast majority of Mdj1 migrated into the gradient, co-localizing with mtDNA. To confirm that Mdj1 is associated with the mitochondrial nucleoid we performed a control experiment in which the mitochondrial extract was treated with DNaseI prior to centrifugation. As expected, such treatment resulted in degradation of mtDNA and a shift of proteins associated with mtDNA (Abf2 and Mdj1) to the top fractions of the sucrose gradient (Suppl. Fig. S2). We also determined the distribution of Ssc1, the Hsp70 partner of Mdj1, as it had previously been reported to be a component of the mitochondrial nucleoid [6]. The majority of Ssc1 remained at the top of the gradient. Only ~ 15% of Ssc1 co-migrated with the nucleoid complex. Taken together our results indicate that most of Mdj1 is associated with the mitochondrial nucleoid.

### 3.2 Depletion of Mdj1 leads to rapid loss of functional mtDNA

**Fig. 2:**
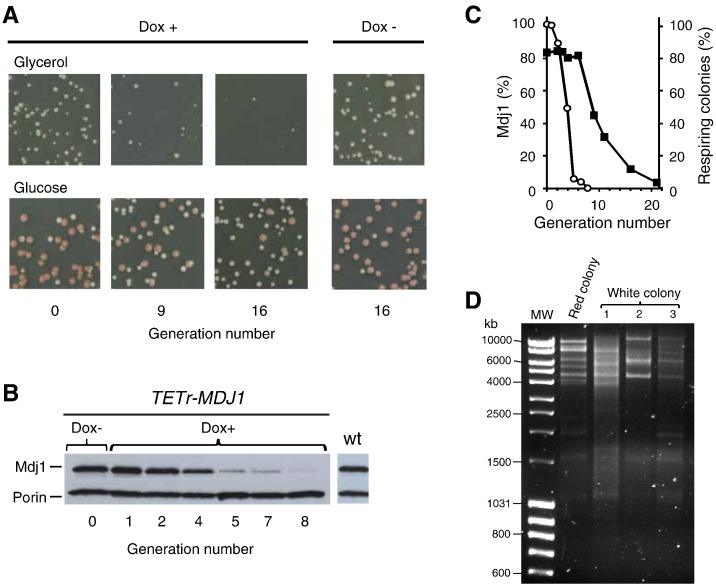
Mdj1 depletion results in loss of functional mtDNA. Doxycycline was added to (Dox +) or, as a control, omitted from a culture (Dox −) of *mdj1*-Δ [*TETr-MDJ1*] cells. At the indicated generations, aliquots of cells were collected and subject to one of three types of analysis: A) plated on either glycerol (upper panel)- or glucose (lower panel)-based media, B) pelleted prior to preparation of cellular extracts, which were subjected to immunoblot analysis or D) used to start subcultures for preparation of mtDNA, which was subjected to restriction endonuclease testing. A) Representative plates from samples harvested at the indicated generations are shown. Red colony color on glucose-based media is an indicator of respiratory competence; white colony color is an indicator of respiratory incompetence. B) Immunoblot analysis of extracts prepared from cells at the indicated number of generations after doxycycline addition, using antibodies specific for Mdj1 and, as a loading control, porin. Also included is an extract of wt cells (wt), as a control to indicate the level of expression of Mdj1. C) Plot of the data obtained. Left Y axis (open circles): the relative amount of Mdj1 obtained by quantitative analysis of results shown in (B), setting the level present prior to the addition of doxycycline at 100%. Right Y axis (closed squares): percentage of cells able to respire, calculated as the ratio of red colonies to total number of colonies (red and white) on glucose containing media. X axis: number of generations of growth in the presence of doxycycline. D) One red colony from a control culture, and three white colonies from a doxycycline treated culture from a 20 generation time point were picked and used to start liquid cultures in glucose-based media. After 12 h at 30 °C total cellular DNA was isolated from each culture. mtDNA was prepared and subjected to digestion with EcoRV, separated by agarose gel electrophoresis, stained with ethidium bromide and visualized under UV light. MW, molecular weight DNA standard Mass Ruler™ DNA Ladder (Fermentas).

The strict requirement of Mdj1 for mtDNA maintenance [12–14], coupled with its substantial nucleoid association, prompted us to begin a more thorough analysis of Mdj1 function in mtDNA maintenance. In the past, the rigid requirement of Mdj1 has been a detriment to analyze Mdj1's role. Therefore, we developed a strain to allow monitoring mtDNA function as Mdj1 is being depleted. The strain we constructed, *mdj1*-Δ [*TETr-MDJ1*], has *MDJ1* under the control of the *TETr* promoter, which allows repression of Mdj1 synthesis upon addition of the drug doxycycline [27,30]. Two methods were used to assess respiratory competence, an indicator of functional mtDNA: (i) the ability of cells to form colonies on media having a nonfermentable carbon source such as glycerol and (ii) colony color on glucose-based media, as respiratory competent cells having a mutation in the *ADE2* gene form red colonies, while respiratory incompetent cells form small white, so-called “petite”, colonies (Fig. 2A).

In the absence of doxycycline, *mdj1*-Δ [*TETr-MDJ1*] cells expressed Mdj1 at a level similar to the wild-type (wt) parental strain (Fig. 2B) and maintained mtDNA as well as wt cells, with 83–85% of cells forming red colonies (Fig. 2A). To test the effect of depletion of Mdj1 on maintenance of mtDNA, drug was added to the culture and cells were collected at intervals, with a portion used to prepare cell extracts for assessing Mdj1 levels. After 4 generations, Mdj1 levels were reduced to ~ 50% of the original; after 8 generations, Mdj1 was undetectable (Fig. 2B). A second portion of the collected cells was used for plating on glycerol-based and glucose-based solid medium lacking drug to assess the functionality of mtDNA. For 6 generations the percentage of respiratory competent cells remained stable (Fig. 2C). However, after that point, respiratory deficient cells accumulated steadily in the population, with ~ 50% of cells being respiratory deficient after 10 generations and over 90% after 20 generations.

Two further controls were carried out. One to test whether the loss of respiratory competence was due to the loss of functional mitochondrial DNA, and secondly, if so, to assess whether the loss was a direct effect of the loss of Mdj1 function in mtDNA transactions *per se* or indirect because of Mdj1 function in the mitochondrial heat shock response and protein folding [23,25]. To test whether loss of respiratory competence upon depletion of Mdj1 actually reflected changes in mtDNA, we characterized the mtDNA present in Mdj1-depleted cells. *mdj1*-Δ cells, like cells lacking other proteins important for functional mtDNA maintenance, first accumulate an array of nonfunctional DNA deletion variants, that is they are rho^−^. They only completely lose mtDNA, that becomes rho^0^, after prolonged culturing [12,13]. mtDNA was isolated from cells obtained from 3 white colonies (Fig. 2D) from the 20th generation time point, and as a control, one rho^+^ red colony from the control culture not treated with doxycycline (Fig. 2D). The isolated mtDNAs were subjected to digestion with a restriction endonuclease and separated by agarose gel electrophoresis. The pattern of digestion of mtDNA isolated from control rho^+^ cells displayed the distinctive pattern of DNA fragments expected [13]. However, typical of rho^−^ DNA, the three experimental samples displayed different patterns of fragments (Fig. 2D).

We next tested whether global protein misfolding occurred upon depletion of Mdj1, thus indirectly affecting mtDNA maintenance. To this end, mitochondria were isolated from two strains, one having wt levels of Mdj1 and one 24 h after initiation of Mdj1 depletion. Protein aggregation was assessed by the standard method of subjecting mitochondrial extracts to glycerol gradient centrifugation to separate aggregated from soluble proteins [38]. No obvious differences in protein profiles were observed between mitochondria having wt levels of Mdj1 and those being depleted of Mdj1 (Suppl. Fig. S3). Protein aggregation was also assessed after shifting of the mitochondria to 48 °C for 20 min. As expected, significant amounts of protein were present in large molecular weight aggregates that pelleted to the bottom of the gradient. However, the profiles of the two mitochondrial extracts were similar, giving no indication of enhanced susceptibility to aggregation of proteins in Mdj1-depleted mitochondria. Together, our data indicate that depletion of Mdj1 *in vivo* results in loss of functional mtDNA, causing respiratory incompetence, and is consistent with the idea that the rapid loss of mtDNA function upon depletion of Mdj1 is a direct effect, rather than an indirect effect due to global protein misfolding.

### 3.3 A functional J-domain is necessary, but not sufficient, for mtDNA maintenance

**Fig. 3:**
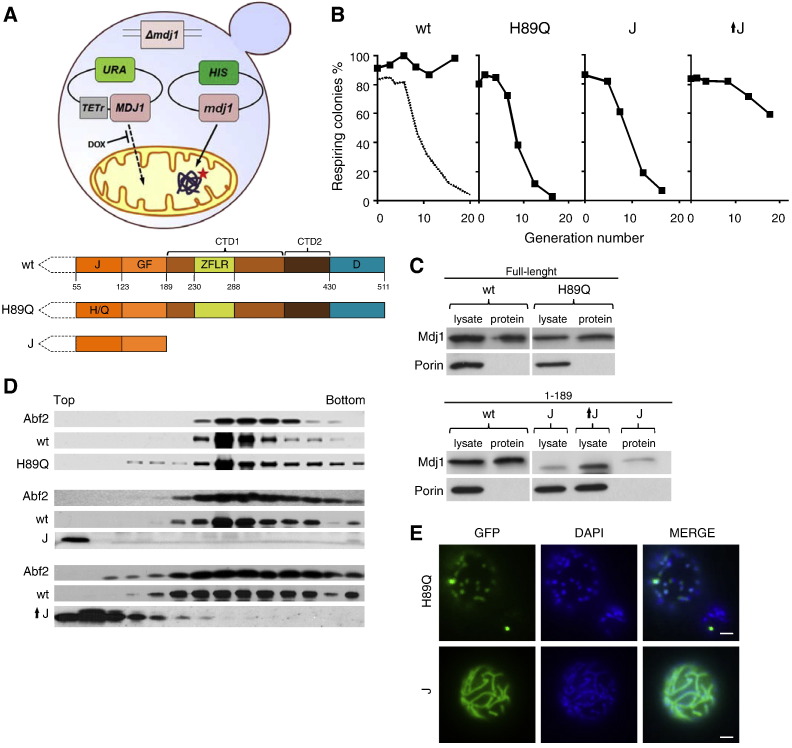
Functional J-domain of Mdj1 is critical for maintenance of mtDNA. A) (Top) Scheme of yeast strain used to analyze the effect of Mdj1 variants on the maintenance of mtDNA upon depletion of wt Mdj1. *mdj1-*Δ [*TETr-MDJ1*] cells were transformed with a plasmid harboring a mutant *mdj1* gene under control of the native *MDJ1* promoter. Since the wt *MDJ1* gene is under the control of the *TETr* promoter, addition of doxycycline (DOX), which represses expression, allows testing of the effect of mutations upon depletion of the wt protein. (Bottom) Scheme of domain composition of Mdj1 variants. N-terminal mitochondrial presequence (dashed line). J-module consists of J-domain (orange) followed by glycine/phenylalanine (GF) -rich linker region (light orange). C-module includes C-Terminal Domain 1 (CTD1; light brown) with extruding Zinc Finger Like Region (ZFLR; green) and C-Terminal Domain 2 (CTD2; brown). D-module is the putative dimerization domain (blue). B) Yeast cells (described in A) expressing the *MDJ1* proteins: as a control, wild-type (wt), the J-domain variant Mdj1_H89Q_ (H89Q) or the J-domain containing fragment Mdj1_Δ190–511_ (J); or overexpressing Mdj1_Δ190–511_ from the *TEF* promoter (↑J) were grown on glucose-based media in the presence of doxycycline. At the indicated number of generations after doxycycline addition aliquots were collected and plated on glucose-based media. The percentage of respiring cells was taken to be the ratio of the number of red colored colonies to the total number of colonies. For comparison, the data from Fig. 2C in which a second plasmid expressing Mdj1 is absent is indicated by a dashed line in the wt plot and thus represents the loss of respiratory competence upon depletion of Mdj1. C) Determination of relative levels of wt Mdj1 and Mdj1 variants in mitochondria. Mitochondrial lysates prepared from cells after growth in the presence of doxycycline for 10 generations, were separated by electrophoresis and subjected to immunoblot analysis using antibodies specific for Mdj1 and, as loading control, porin. Because the polyclonal antibody used reacts differently with wt and truncated forms of Mdj1, 0.2 pmol of purified wt Mdj1 or indicated variants was run on the same gel (protein), as a semi-quantitative reference for expression. Slower migration of the purified J variant is due to the presence of 6 His tag at the C-terminus. D) Distribution of Mdj1 variants after centrifugation of mitochondrial lysates. Mitochondrial lysates isolated from *MDJ1*/*mdj1*-Δ diploid cells expressing the Mdj1 variants Mdj1_H89Q_ (H89Q) and (J) under control of native *MDJ1* promoter or overexpressed (↑J) under control of *TEF* promoter, grown in glucose-based medium were subjected to ultracentrifugation through a sucrose gradient. Each fraction was analyzed for protein content using immunoblot analysis with antibodies specific for Mdj1 or, as a control, Abf2. In the case of Mdj1_H89Q_, a fusion with GFP was used to allow separation from wt Mdj1 during electrophoresis. E) Cellular localization of Mdj1 variants. *MDJ1*/*mdj1*-Δ diploid cells constitutively expressing GFP fusions of Mdj1_H89Q_ (H89Q) or Mdj1_Δ190–511_ (J) were analyzed by fluorescence microscopy. Cellular DNA was stained with DAPI. Overlay of the two images (MERGE). Size bars (2 μm) are shown.

Using the assay described above, we began an assessment of the regions of Mdj1 required for mtDNA maintenance. To carry out such tests, a low copy number plasmid encoding the variant to be tested under the control of the native *MDJ1* promoter was transformed into *mdj1-*Δ [*TETr-MDJ1*] (Fig. 3A). In the absence of drug, both wt Mdj1 and the variant are co-expressed, but after addition of drug to repress expression from the *TETr* promoter, the vast majority of Mdj1 present is the variant, allowing testing of its ability to function in maintaining respiratory competence. Using this system, we first asked if the function of the J-domain of Mdj1 is critical for maintenance of mtDNA. We utilized a variant having an alteration in the conserved, J-domain HPD motif [18], in which the histidine was replaced by a glutamine (H89Q) (Fig. 3A). Like H to Q variants of other J-proteins previously tested [18], Mdj1_H89Q_ is not functional, even though it is expressed at levels equivalent to wt (Fig. 3C). We found that respiratory competence was lost with kinetics which are very similar to those found in the test strain having wt *MDJ1* under the control of the *TETr* promoter as the only *MDJ1* gene (Fig. 3B, Suppl. Fig. S4). On the other hand, when, as a positive control, wt Mdj1, rather than Mdj1_H89Q_, was expressed from the *MDJ1* promoter, cells remained respiratory competent, as expected (Fig. 3B). These results indicate that functional, J-domain dependent, cooperation with Ssc1 as part of the Hsp70 chaperone machinery is critical for maintenance of mtDNA.

The functions of several J-proteins, including Ydj1 of the yeast cytosol and DnaJ of *E. coli*, can be performed by fragments containing their J-domains and adjusted glycine/phenylalanine (GF)-rich region, but lacking other regions, including those important for client protein binding [39,40]. Therefore, we decided to ask whether this is also the case for Mdj1's function in mtDNA maintenance. To this end we constructed a *MDJ1* truncation mutant that encodes a fragment lacking the C-terminal 321 amino acids, which after cleavage of the presequence upon entering the mitochondrial matrix, generates a 134 amino acid fragment having the J-domain at the N-terminus (Fig. 3A). After addition of drug, to repress expression of full-length Mdj1, we observed a rapid loss of respiratory capacity of cells in the culture (Fig. 3B, Suppl. Fig. S4), similar to that observed in the control strain. To make sure that the short J-domain fragment was expressed at the level comparable to the full-length protein we decided to compare their concentrations in cellular extracts utilizing immunoblot using polyclonal antibodies against Mdj1. We noticed however, that the signal obtained for the small J-domain fragment was much weaker than that obtained for full length Mdj1 protein (Fig. 3C) because the polyclonal antibody used reacted with different efficiency with full length and truncated forms of Mdj1. Therefore, we standardized the immunoblot signals by comparing them to those obtained for equivalent amounts of purified proteins run on the same gel. Based on this analysis we concluded that full-length Mdj1 and the J-domain fragment were expressed at similar levels (Fig. 3C).

Next we assessed whether the two Mdj1 variants were nucleoid-associated. We expressed either Mdj1_H89Q_ or the J-domain fragment in a diploid heterozygous strain in which one copy of *MDJ1* was intact to ensure the functional integrity of the nucleoid (*MDJ1*/*mdj1*-Δ). We subjected mitochondrial extracts from cells expressing full-length Mdj1, Mdj1_H89Q_ or the J-domain fragment to centrifugation analysis. The J-domain fragment remained at the top of the gradient. However, like wt Mdj1 and the control protein Abf2, Mdj1_H89Q_ migrated into the middle, consistent with mitochondrial nucleoid localization (Fig. 3D). However, as Hsp40s are known to function as dimers, we performed an additional control to make sure that the possible formation of the Mdj1/Mdj1_H89Q_ heterodimers was not responsible for the nucleoid localization of the Mdj1_H89Q_. To this end, we repeated the localization experiment utilizing Mdj1_H89Q∆D_ variant lacking the putative dimerization domain. Since this variant co-localized with the nucleoid (Suppl. Fig. S5) we concluded that indeed replacement of H89Q did not affect nucleoid localization of the Mdj1_H89Q_ protein.

Moreover, the J-domain fragment fused to GFP showed a diffuse pattern of fluorescence (Fig. 3E), similar to that of mtGFP (Fig. 1A). On the other hand, Mdj1_H89Q_-GFP showed a punctated pattern (Fig. 3E), similar to Mdj1 and typical for established mitochondrial nucleoid localized proteins such as Abf2 (Fig. 1A). To ask directly whether the J domain is required for nucleoid association we created a variant, Mdj1_∆J_ that lacks the entire J domain (residues 55 to 123). Similar to Mdj1_H89Q_, Mdj1_∆J_ localized to the nucleoid complex and, as expected was unable to maintain mtDNA (Suppl. Figs. S4 and S6). We conclude that the J-domain is not critical for nucleoid association and that the J-domain fragment, which is unable to support maintenance of mtDNA, is not associated with the mitochondrial nucleoid.

Since the J-domain fragment was not nucleoid-associated, we tested whether it could function in maintenance of mitochondrial DNA when overexpressed. We placed this truncation under the control of the highly expressed *TEF* promoter, which resulted in approximately 5 fold higher levels than when its expression was driven from its own promoter (Fig. 3C). When expressed at this level, mtDNA was lost upon repression of expression of full-length Mdj1. However, the rate of loss was slower than when the fragment was expressed at normal levels. Thus, at high concentrations the J-domain fragment alone is partially functional in mtDNA maintenance.

### 3.4 CTD1/2 is critical for maintenance of mtDNA and localization to the mitochondrial nucleoid

**Fig. 4:**
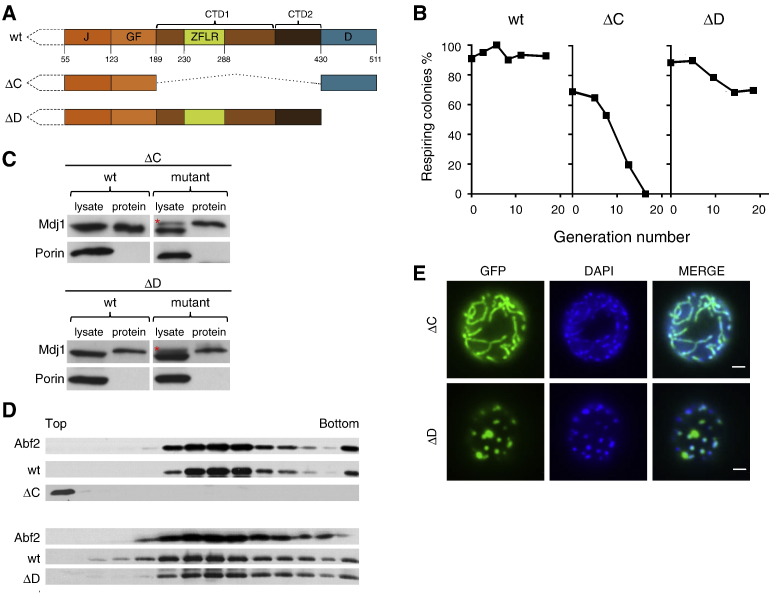
CTD1/2 domains of Mdj1 are critical for maintenance of mtDNA. A) Scheme of domain composition of Mdj1 deletion variants as in Fig. 3A. Internal deletion of Mdj1_ΔC_ is indicated by dotted line. B) *mdj1-*Δ [*TETr-MDJ1*] expressing Mdj1 (wt), Mdj1_ΔC_ (∆C) or Mdj1_ΔD_ (∆D) under control of the *MDJ1* promoter was grown in the presence of doxycycline. At the indicated number of generations after doxycycline addition, aliquots were collected and plated on glucose-based media. The percentage of respiring cells was taken as the ratio of the number of red colored *versus* the total number of colonies. C) Mitochondrial lysates were prepared from cells harvested 10 generations after doxycycline addition and subjected to immunoblot analysis using antibodies specific for Mdj1 and, as loading control, porin. As a quantity reference 0.2 pmol of purified wt Mdj1 (wt), Mdj1_ΔC_ (∆C) or Mdj1_ΔD_ (∆D), was run on the same gel (protein), as the polyclonal Mdj1 antibody does not recognize Mdj1, Mdj1_ΔC_ and Mdj1_ΔD_ with the same efficiency. Slower migration of the purified ΔC and ΔD variants is due to the presence of 6 His tag at the C-terminus. Red star indicates unspecific bands. D) Distribution of Mdj1 variants after centrifugation of mitochondrial lysates. Mitochondrial lysates isolated from *MDJ1*/*mdj1-Δ* diploid cells expressing wt Mdj1 (wt) or the variants, Mdj1_ΔC_ (∆C) or Mdj1_ΔD_ (∆D) were subjected to ultracentrifugation through a sucrose gradient. Each fraction was analyzed for protein content using immunoblot analysis with antibodies specific for Mdj1 or, as a control, Abf2. E) Cellular localization of Mdj1 variants. Cells expressing GFP fused to Mdj1_ΔC_ (∆C) or Mdj1_ΔD_ (∆D) were analyzed by fluorescence microscopy. Cellular DNA was stained with DAPI. Overlay of the two images (MERGE). Size bars (2 μm) are shown.

To begin to narrow down the region of Mdj1 important for nucleoid localization and mtDNA maintenance, we first tested two deletion variants: (i) Mdj1_∆C_, lacking the C-terminal domains, CTD1and CTD2, but maintaining the putative dimerization domain and (ii) Mdj1_∆D_, lacking the C-terminal 81 amino acids of this dimerization domain (Fig. 4A). Both were expressed at levels similar to normal Mdj1 levels (Fig. 4C). Cells expressing the ΔC allele lost respiratory competence with kinetics similar to that observed for Mdj1_H89Q_ (compare Fig. 4B with Fig. 3B, Suppl. Fig. S4). Cells expressing Mdj1_∆D_ also showed a decrease in respiratory competence. However, this loss was significantly slower than that of cells expressing either Mdj1_H89Q_ or Mdj1_∆C_, as 70% of the cells were competent 20 generations after the addition of drug (Fig. 4B, Suppl. Fig. S4). This observation is consistent with the putative dimerization domain, being important, but not critical for Mdj1's role in the maintenance of mtDNA. Next, we tested the nucleoid association of these two variants. Mdj1_∆D_, but not Mdj1_∆C_, was associated with the nucleoid, as judged both by sucrose gradient centrifugation and microscopic observations of GFP fusions (Fig. 4D, E). Thus, the presence of CTD1 and CTD2 domains, but not the dimerization domain, is critical for both maintenance of functional mtDNA and mitochondrial nucleoid association.

### 3.5 Zn finger-like region and the peptide-binding cleft are not critical for mtDNA maintenance or mitochondrial nucleoid localization

**Fig. 5:**
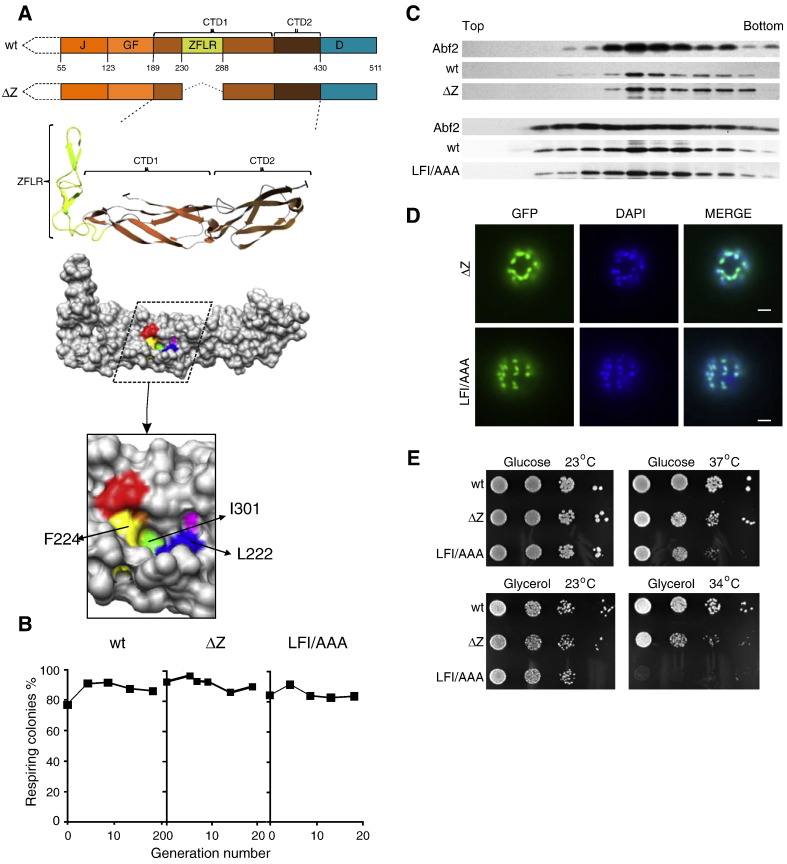
Neither deletion of zinc finger like region nor alteration of residues in substrate-binding cleft affects maintenance of mtDNA. A) (Top) Scheme of Mdj1_ΔZ_ with dotted line indicating the deletion of the zinc finger like region (for details see Fig. 3A). (Bottom) Structural model of C module with ZFLR, CTD1 and CTD2 indicated and with residues of the substrate binding cleft red — Ile^202^, blue — Leu^222^, yellow — Phe^224^, green — Ile^301^, orange — Ile^343^, magenta — Val^345^. Arrows indicate residues, which were altered in this study. B) *mdj1-*Δ [*TETr-MDJ1*] expressing Mdj1 (wt), Mdj1_ΔZ_, Mdj1_LFI/AAA_ under the control of the *MDJ1* promoter was grown in the presence of doxycycline. At the indicated number of generations after doxycycline addition, aliquots were collected and plated on glucose-based media. The percentage of respiring cells was determined as the ratio of the number of red colored *versus* the total number of colonies on plates. C) Distribution of Mdj1 variants after centrifugation of mitochondrial lysates. Mitochondrial lysates isolated from *MDJ1*/*mdj1*-Δ diploid cells expressing Mdj1 (wt) or the Mdj1 variants, Mdj1_ΔZ_, or Mdj1_LFI/AAA_ were subjected to ultracentrifugation through a sucrose gradient. Each fraction was analyzed for protein content using immunoblot analysis with antibodies specific for Mdj1 or, as a control, Abf2. Mdj1_LFI/AAA_ GFP fusions were used to allow separation from wt Mdj1 during electrophoresis. D) Cellular localization of Mdj1 variants. Cells expressing GFP fusions of Mdj1_ΔZ_ (∆Z-GFP) or Mdj1_LFI/AAA_ (LFI/AAA-GFP) were analyzed by fluorescence microscopy. Cellular DNA was stained with DAPI. Overlay of the two images (MERGE). Size bars (2 μm) are shown. E) *mdj1*-Δ cells harboring plasmid-borne copies of wt *MDJ1*, ΔZ or LFI/AAA variants, as indicated, were plated as 10-fold dilutions on glucose-rich medium (top) or glycerol-rich medium (bottom) and incubated at indicated temperatures for 3 days (glucose) or 4 days (glycerol).

Two features within the region encompassing CTD1 and CTD2 have been defined structurally and functionally, the zinc finger-like region (ZFLR) and the peptide binding cleft (Fig. 5A) [20–22]. To assess their importance in maintenance of mtDNA, we constructed two types of *MDJ1* mutations: (i) a complete deletion of the ZFLR (Mdj1_∆Z_) and (ii) alterations of hydrophobic amino acids in the peptide-binding cleft (Mdj1_LFI/AAA_) (Fig. 5A). The respiratory competence of Mdj1_∆Z_ and Mdj1_LFI/AAA_ cells remained constant after depletion of wt Mdj1 (Fig. 5B), indicating that neither the zinc finger-like region nor the peptide cleft is critical for maintenance of mtDNA. Both sucrose gradient analysis of mitochondrial extracts and microscopic evaluation indicated that these variants were nucleoid-associated (Fig. 5C, D). We also tested the growth phenotype of a strain having *mdj1*_∆*Z*_ or *mdj1*_*LFI*/*AAA*_ as the only copy of the *MDJ1* gene (Fig. 5E). Both *mdj1*_∆*Z*_ and *mdj1*_*LFI*/*AAA*_ grew on glycerol based medium, indicating mtDNA function. However, they grew more slowly at 37 °C on rich glucose-based media, with *mdj1*_*LFI*/*AAA*_ barely able to form colonies (Fig. 5E). This temperature sensitive growth is consistent with client protein binding being needed for growth at borderline temperatures, perhaps in general protein folding. However, our data provides no evidence that either of the two well-defined features of the region encompassing CTD1 and CTD2 is necessary for mtDNA maintenance.

### 3.6 DNA binding ability of Mdj1 variants correlates with their mitochondrial nucleoid association

**Fig. 6:**
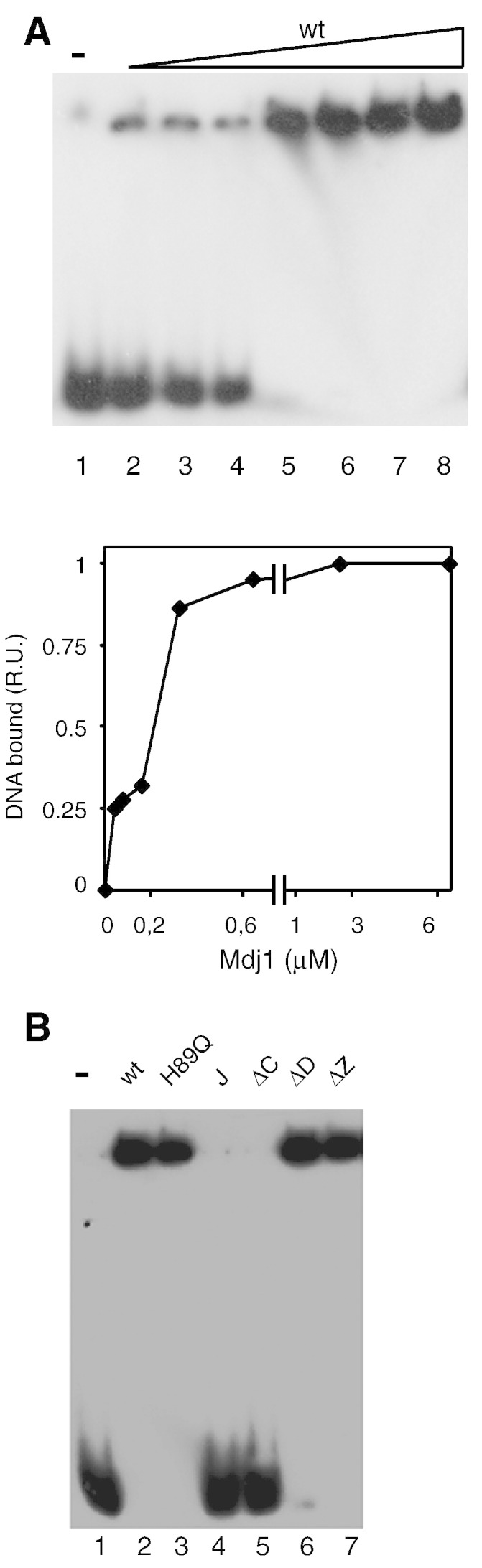
Mdj1 binds DNA *in vitro*. A,B) Gel mobility shift analysis of Mdj1 binding to an mtDNA fragment. Purified Mdj1 or its variants were incubated with ^32^P-labeled 71 bp *ori5* fragment at 0.36 nM, and then subjected to electrophoresis, followed by autoradiography. A) (Top) Different concentrations of wt Mdj1 (wt): Lines 1–8: at concentrations: 0 (indicated by “−”); 0.04, 0.08, 0.16, 0.32, 0.64, 2.56 and 6.4 μM, respectively. (Bottom) Quantification of the results (apparent Kd ~ 0.21 μM). B) Mdj1 variants at 0.64 μM were analyzed: (−) no protein control; (wt) wild-type; (H89Q) Mdj1_H89Q_; (J) J-domain fragment Mdj1_Δ190–511_; (ΔC) Mdj1_Δ201–429_; (ΔD) Mdj1_Δ430–511_ ΔZ) Mdj1_Δ230–288_.

It was previously reported that bacterial homologs of Mdj1 are able to directly interact with DNA in a sequence nonspecific manner [41,42]. Since we had found no evidence that either the peptide binding cleft or the zinc finger-like region was important for either mtDNA maintenance or nucleoid association, we decided to test whether Mdj1 also possesses DNA binding ability and, if so, whether it might be important for nucleoid association. To test for Mdj1-DNA interaction, we used a band shift assay using a radio-labeled short fragment of DNA (71 bp) derived from a mitochondrial DNA origin, *ori5*. At higher Mdj1 concentrations, the DNA fragment shifted to the top of the gel, indicating that Mdj1 does bind DNA *in vitro* (Fig. 6A). This binding appears to be nonspecific, as Mdj1 also bound DNA fragments derived from sources other than mtDNA (data not shown). To determine whether DNA binding ability correlated with the association of Mdj1 with the nucleoid, we tested the Mdj1 variants described in the sections above in the DNA binding assays, with the exception of the variants having alterations in the peptide-binding cleft as they were prone to aggregation. The variants were lacking the zinc finger-like region (Mdj1_∆Z_), lacking the dimerization domain (Mdj1_∆D_) or carrying the inactive H89Q J-domain bound DNA. However, Mdj1_∆C_ and Mdj1_Δ190–511,_ the larger deletion retaining only the J-domain and glycine-phenylalanine rich (GF) region, did not (Fig. 6B). Thus, the results of *in vitro* DNA binding tests correlated with the results of *in vivo* nucleoid localization, as variants able to bind DNA were found associated with the mitochondrial nucleoid, whereas variants unable to bind DNA were found in the fractions having soluble proteins.

## 4 Discussion

The results reported here not only indicate that Mdj1's roles in mtDNA maintenance require functional cooperation with its Hsp70 partner, Ssc1, but that the vast majority of Mdj1 is localized to the nucleoid. Our data also suggests that Mdj1's function in mtDNA maintenance is facilitated by this tethering, which may occur *via* interaction with DNA.

### 4.1 Association of Mdj1 with the nucleoid correlates with DNA binding and mtDNA maintenance

Both microscopic observations and biochemical fractionation experiments demonstrate that the majority of Mdj1 is associated with the mitochondrial nucleoid. Such concentration at the nucleoid is somewhat unexpected, as its distribution is more similar to that of mtDNA packaging protein such as Abf2 than that of other chaperones or metabolic enzymes that are well-established constituents of the mitochondrial nucleoid [1,35,43]. For example, only a minor fraction of the mitochondrial content of Aco1, aconitase, and Ilv5, an enzyme involved in branched-chain amino acid biosynthesis, is found associated with the nucleoid, even though, like Mdj1, they bind DNA nonspecifically [35,43]. Such concentrated localization points to an important role of Mdj1 at the mitochondrial nucleoid.

Our results are consistent with Mdj1's nucleoid localization being due to direct binding to DNA. We found no evidence that known structural features of CTD1/2, such as the zinc finger like region and the peptide binding cleft, are necessary for either DNA binding or nucleoid association. However, for each variant tested, *in vitro* DNA binding ability and association with the nucleoid complex *in vivo* correlated. Interestingly, a J-protein of *E. coli*, CbpA, has been reported to be associated with the nucleoid and to bind double-stranded DNA nonspecifically [41,42]. No biological role of CbpA associated with the nucleoid has been found. However, *in vitro* results reported for CbpA are similar to those we have obtained with Mdj1 [42]. Alterations of amino acids responsible for interaction with protein substrate did not affect CpbA DNA binding activity, suggesting, like the results reported here, that DNA binding is independent of peptide binding. Clearly, further studies are needed to determine the residues responsible for DNA binding, the biochemical nature of Mdj1-DNA interaction and how this interaction enables Mdj1 to associate with the mitochondrial nucleoid complex.

### 4.2 Mdj1's co-operation with Hsp70 is critical for mtDNA maintenance

The stringency of the requirement of the J-domain in maintenance of mtDNA is striking. Upon depletion of wt Mdj1 from cells, the rate of loss of respiratory capacity of variants lacking J-domain function mirrored that of cells expressing no Mdj1, even though these variants showed normal association with nucleoids. Thus, we conclude, that Mdj1's function in maintaining functional mtDNA requires its co-chaperone activity as part of the Hsp70 chaperone system. However, when expressed at normal levels, the J-domain is not sufficient. The association of Mdj1 with the nucleoid results in a high local concentration of Mdj1, which serves to recruit its Hsp70 partner to the nucleoid. This idea is consistent with overexpression of the non-nucleoid associated J-domain supporting mtDNA maintenance. Rescue of the lack of J-protein function in *E. coli* by over-expression of a J-domain fragment is well documented in the case of the bacterial ortholog of Mdj1, DnaJ [40]. Such rescue by overexpression of the J-domain fragment is also consistent with the idea that direct binding of Mdj1 to specific clients is not a critical feature of its role at the mitochondrial nucleoid.

### 4.3 Role(s) of Mdj1 in mtDNA transactions

The results presented here, along with previously published results [12–14], underscore the fact that Mdj1 is critical for maintenance of mitochondrial DNA transactions. More work is needed to pinpoint the specific role(s) Mdj1 plays in maintaining the fidelity of mtDNA replication and transmission. However, together the data point to multiple roles, some constitutive, others important only under stress conditions.

Under stress conditions, such as after a heat shock and during growth at borderline temperatures, evidence supports a role of Mdj1 in the maintenance of correctly folded conformations of proteins. Mdj1, together with Ssc1, is involved in *de novo* protein folding and in protection of mitochondrial proteins against heat stress initiated protein unfolding [12,23–25]. Particularly germane to this discussion, Mdj1 plays a critical role in the protection of the enzymatic activity of mitochondrial DNA polymerase, Mip1, upon extreme heat stress conditions [13,24]. Mitochondrial polymerase was immuno-precipitated with the mammalian ortholog of Mdj1, DnajA3/Tid1, supporting a view that mitochondrial DNA polymerase is a native substrate of Mdj1 [11]. It is important to stress, however, that under optimal growth conditions the biochemical activity of mitochondrial DNA polymerase Mip1 is not affected by deletion of *MDJ1* [13].

Since all of the evidence for Mdj1's involvement in protein folding has come from experiments carried out under heat shock conditions, it is tempting to speculate that its role in maintenance of mtDNA goes beyond assistance in folding or refolding of proteins. We suggest that Mdj1 may play a more direct role in promoting formation or rearrangement of protein complexes involved in the maintenance and propagation of mitochondrial genomes. Although direct targets of Mdj1 in remodeling of protein complexes have yet to be identified, the involvement of homologous J-proteins in the rearrangement of protein complexes in the process of DNA replication is well documented in viral replication. In the case of bacteriophage λ, a J-protein having similar architecture to that of Mdj1, DnaJ, and its Hsp70 partner DnaK acts together to dissociate the inhibitory protein λP from the pre-primosomal complex, thus activating DNA helicase, a requirement for progression of λDNA replication [44]. Hsp70 machinery is also involved in the activation of proteins necessary for SV-40 DNA replication. Large T-antigen, a virus encoded protein responsible for initiation of the viral DNA replication, contains its own J-domain, which recruits a host Hsp70. Similar to the bacterial case, the chaperones facilitate dissociation of a protein complex, a requirement for activation of viral DNA replication [45].

It is important to stress that mitochondrial nucleoid proteins are involved, not only in mtDNA replication, but also in transcription and translation of mitochondrial genes [4]. In yeast, and likely in other organisms as well, disruption of any of these processes leads to destabilization of the mitochondrial genome and its eventual loss [4]. Therefore, potential targets of the Mdj1/Ssc1 machinery could be proteins participating in transcription or translation, as well as those directly involved in mtDNA replication *per se*. Interestingly, J-domain dependent functions of large T antigen extend beyond DNA replication to, for example, regulation of transcription of viral and host genes [45]. Further work is clearly needed both to identify molecular targets and mechanisms responsible for the essential role(s) of the Mdj1/Ssc1 chaperone machinery localized to the nucleoid in maintenance and propagation of mitochondrial genomes and to understand the functional implications of localization of the vast majority of Mdj1 to the nucleoid, which leaves the rest of the matrix with a low concentrations of this J-protein.
