# Supplementary material for: Nucleoid localization of Hsp40 Mdj1 is important for its function in maintenance of mitochondrial DNA
Source: Biochim Biophys Acta. 2013 Oct;1833(10):2233–43. doi: 10.1016/j.bbamcr.2013.05.012 (PMC3750215; doi:10.1016/j.bbamcr.2013.05.012)

SUPPLEMENTARY MATERIAL

Supplementary Figure S1. Mdj1-GFP fusion protein is functional in vivo.

A) 10-fold dilutions of cells were plated on rich media having either glucose or glycerol as a carbon source, as indicated, and incubated at the indicated temperatures for 3 days (glucose) or 4 days (glycerol). Strains: wild-type harboring plasmid borne copy of Mdj1-GFP fusion protein (wt+Mdj1-GFP), wild-type (wt), *mdj1-Δ* (*Δmdj1*), *mdj1-Δ* harboring plasmid-borne copy of Mdj1-GFP (*Δmdj1* + Mdj1-GFP).
B) Cell lysates prepared from strains used in (A) were subjected to immunoblot analysis using antibodies specific for Mdj1 and, as loading control, porin. lane 1- wt+Mdj1-GFP; lane 2-wt; lane 3-*Δmdj1*; lane 4-*Δmdj1* + Mdj1-GFP

Supplementary Figure S2. Distribution of Mdj1 after centrifugation of mitochondrial lysates pre-treated with DNaseI.

Lysate prepared from isolated mitochondria was incubated with DNaseI (at 10U/50 μg of protein) for 60 min. in the cold-room with rotation before it was subjected to ultracentrifugation through a sucrose gradient (as described in "Materials and Methods"). A portion of each fraction was analyzed for mtDNA content by PCR amplification of mtDNA *ori5* fragment (mtDNA) and for protein content by immunoblot analysis using antibodies specific for Mdj1and Abf2 as indicated.

Supplementary Figure S3. Depletion of Mdj1 does not result in global protein misfolding in mitochondria from cells grown at optimal temperature.

A, B) *mdj1*-Δ [*TETr-MDJ1*] expressing Mdj1 (wt) or harboring empty plasmid were grown at 30°C in the presence of doxycycline for 24 h to allow depletion of Mdj1 controlled by the *TETr* promoter. Isolated mitochondria (1 mg protein) were resuspended in buffer SG (200 μl) and either keep on ice (A) or incubated at 48°C for 20 minutes (B). Mitochondria isolated from isogenic strain lacking mtDNA (rho^{o}) were used as a control, as indicated. Following lysis in the presence of 0.5% (v/v) NP-40, 1 mM PMSF and 3 mM spermidine, samples were loaded onto 3 ml of 15-40% (v/v) glycerol gradient prepared in buffer SG and centrifuged in a Beckman SW60Ti rotor for 2 h at 46,000 rpm at 4°C; fractions were collected from the top of the gradient. The protein pellet from the bottom of the tube was solubilized by incubation with a Laemmli sample buffer (Tris-HCl 60 mM pH 6.8, glycerol 20%, sodium dodecyl sulfate 2%, β-mercaptoethanol 5%, bromophenol blue 0.01%) at 95 °C. Equivalent amounts of fractions were separated by SDS-PAGE following by Coomassie Blue G staining.

Supplementary Figure S4. Growth phenotypes of yeast mdj1-Δ cells harboring plasmid borne copies of mdj1 mutants as the only copy of the MDJ1 gene.

Indicated strains were plated as10-fold dilution series on glucose-or glycerol-rich medium and incubated at 30 °C for 3 (glucose) or 4 (glycerol) days. *mdj1-Δ* cells harboring plasmid-borne copies of: wt Mdj1 [wt]; empty vector [Vect]; $Mdj1_{H89Q}$ [H89Q]; $Mdj1_{ΔJ}$ [ΔJ]; $Mdj1_{ΔC}$ [ΔC]; J-domain containing fragment $Mdj1_{Δ190\text{-}511}$ [J]; or $Mdj1_{Δ190\text{-}511}$ under the control of the *TEF* promoter [↑J]; $Mdj1_{ΔZ}$ [ΔZ]; $Mdj1_{ΔD}$ [ΔD].

Supplementary Figure S5. Lack of putative dimerization domain does not affect nucleoid localization of $Mdj1_{H89Q}$ and $Mdj1_{ΔZ}$ variants.

A) Scheme of the dimerization domain lacking $Mdj1_{H89QΔD}$ and $Mdj1_{ΔZΔD}$variants. N-terminal mitochondrial presequence (dashed line; aa 1-55). J-module consists of J-domain (orange; aa55-123) followed by glycine/phenylalanine (GF) -rich linker region (light orange; aa 124-189). C-module includes C-Terminal Domain 1 (CTD1; light brown; aa 190-350) with extruding Zinc Finger Like Region (ZFLR; green; aa 230-288) and C-Terminal Domain 2 (CTD2; brown; aa 363-429). D-module is the putative dimerization domain (blue; aa 430-511). Internal deletion of $Mdj1_{ΔZ}$ is indicated by dotted line. B) Distribution of $Mdj1_{H89QΔD}$ after centrifugation of mitochondrial lysate. Mitochondrial lysates isolated from MDJ1/mdj1-Δ diploid cells expressing $Mdj1_{H89QΔD}$ were subjected to ultracentrifugation through a

sucrose gradient. Each fraction was analyzed for protein content using immunoblot analysis with antibodies specific for Mdj1 or, as a control, Abf2. C) Distribution of Mdj1$_{\Delta Z \Delta D}$ after centrifugation of mitochondrial lysate (see B for details).

Supplementary Figure S6. Presence of J-domain of Mdj1 is critical for maintenance of mtDNA.

A) Scheme of the J-domain lacking Mdj1$_{\Delta J}$ variant. N-terminal mitochondrial presequence (dashed line; aa 1-55). J-module consists of J-domain (orange; aa55-123) followed by glycine/phenylalanine (GF) -rich linker region (light orange; aa 124-189). C-module includes C-Terminal Domain 1 (CTD1; light brown; aa 190-350) with extruding Zinc Finger Like Region (ZFLR; green; aa 230-288) and C-Terminal Domain 2 (CTD2; brown; aa 363-429). D-module is the putative dimerization domain (blue; aa 430-511). Internal deletion of Mdj1$_{\Delta J}$ is indicated by dotted line. B) *mdj1*-Δ [*TETr-MDJ1*] expressing Mdj1 (wt), Mdj1$_{\Delta J}$ under control of the *MDJ1* promoter were grown in the presence of doxycycline. At the indicated number of generations after doxycycline addition, aliquots were collected and plated on glucose-based media. The percentage of respiring cells was taken as the ratio of the number of red colored versus the total number of colonies. C) Distribution of Mdj1$_{\Delta J}$ after centrifugation of mitochondrial lysate. Mitochondrial lysates isolated from *MDJ1/mdj1-Δ* diploid cells expressing Mdj1$_{\Delta J}$ were subjected to ultracentrifugation through a sucrose gradient. Each fraction was analyzed for protein content using immunoblot analysis with antibodies specific for Mdj1 or, as a control, Abf2. D) Cellular localization of Mdj1 variants. Cells expressing GFP fused to Mdj1$_{\Delta J}$ were analyzed by fluorescence microscopy. Cellular DNA was stained with DAPI. Overlay of the two images (MERGE). Size bar (2 μm) is shown.

Supplementary figure S1

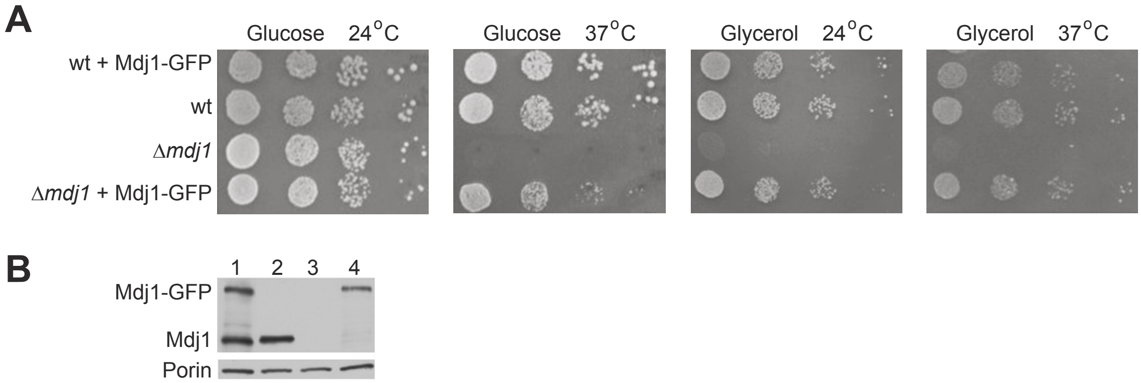

Supplementary figure S2

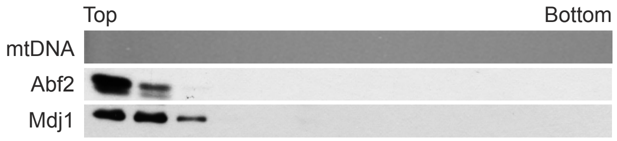

Supplementary figure S3

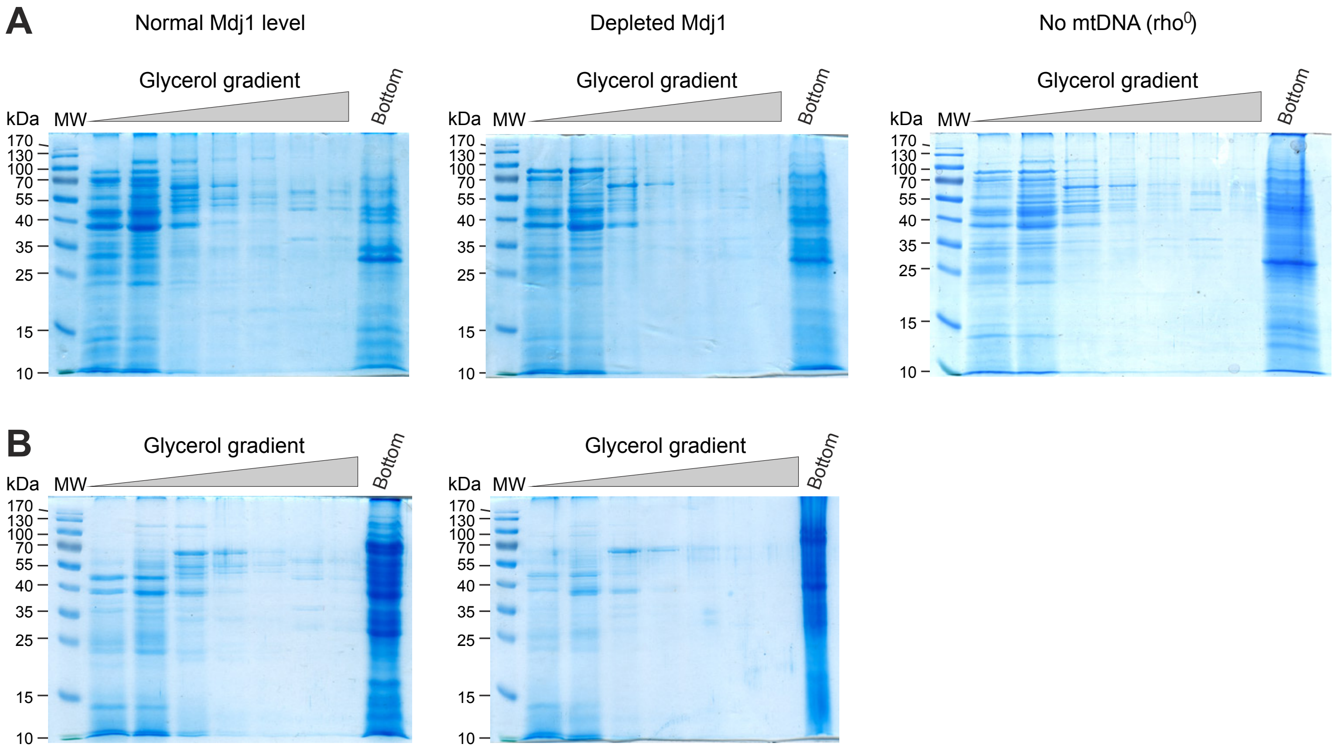

Supplementary figure S4

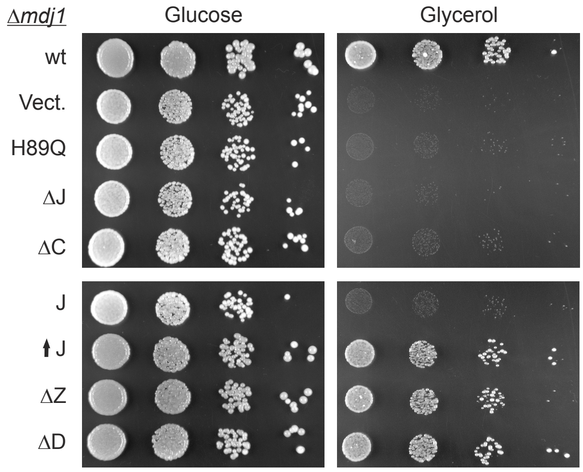

Supplementary figure S5

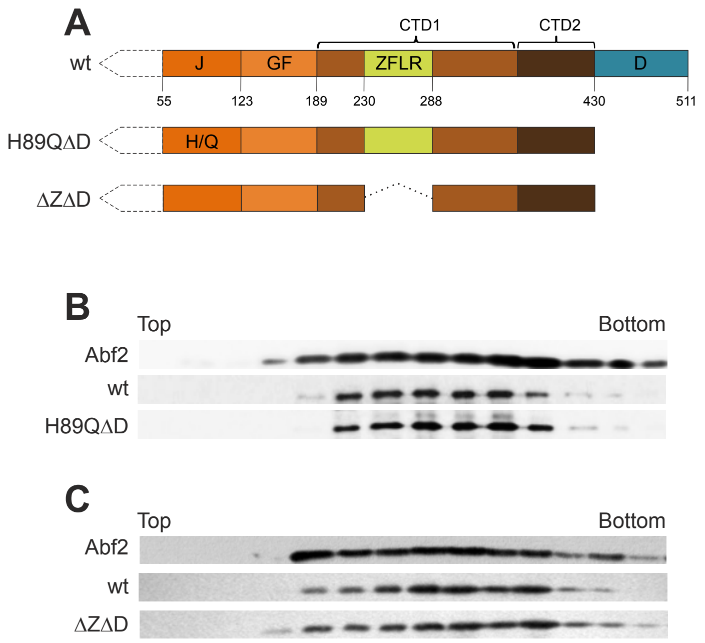

Supplementary figure S6

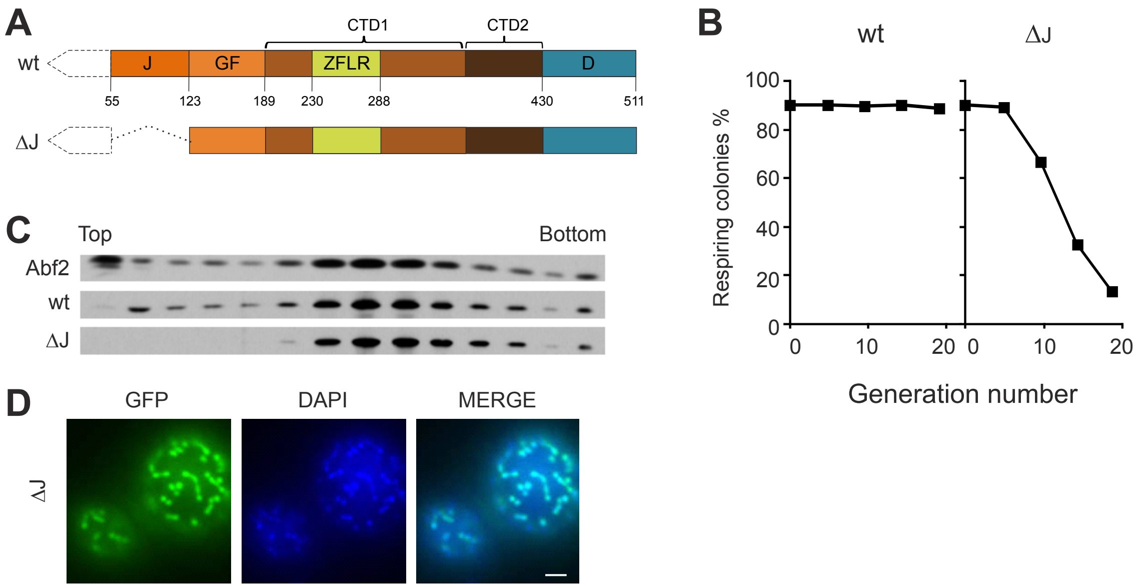

Supplement: Supplementary file 1 — Supplementary figures. [file mmc1.pdf]
